# Comparison of lens refractive parameters in myopic and hyperopic eyes of 6–12-year-old children

**DOI:** 10.3389/fmed.2022.942933

**Published:** 2022-12-08

**Authors:** Jianming Shang, Yanjun Hua, Yuliang Wang, Ji C. He, Xingtao Zhou, Xiaomei Qu

**Affiliations:** ^1^Department of Ophthalmology and Vision Science, Eye & ENT Hospital, Fudan University, Shanghai, China; ^2^NHC Key Laboratory of Myopia, Fudan University, Shanghai, China; ^3^Laboratory of Myopia, Chinese Academy of Medical Sciences, Shanghai, China; ^4^Department of Ophthalmology, Shanghai Jiao Tong University Affiliated Sixth People's Hospital, Shanghai, China; ^5^New England School of Optometry, Boston, MA, United States

**Keywords:** lens curvature, lens refractive power, cycloplegia, pathogenesis of myopia, hyperopia

## Abstract

**Background/aims:**

To evaluate the influence of cycloplegia on lens refractive parameters in 6–12-year-old children with myopia and hyperopia for exploring the pathogenesis of myopia.

**Methods:**

One hundred eyes of 100 patients (50 boys) were included. In the myopic group, 50 subjects (25 boys and 25 right eyes) were enrolled with a mean age of 9.20 ± 1.69 years. IOLMaster 700 measurements were performed pre- and post-cycloplegia. The pictures were marked using semi-automatic software. The lens curvature and power were obtained using MATLAB image processing software. Paired and independent sample *t*-tests were used for data analysis. Statistical significance was set at *P* < 0.05.

**Results:**

Anterior and posterior lens curvature radius in myopic eyes were larger than those in hyperopic eyes, both pre- and post-cycloplegia (both *P* < 0.001). The refractive power in myopic eyes was lower than that in hyperopic eyes without cycloplegia, both pre- and post-cycloplegia (both *P* < 0.001). The changes in anterior lens curvature and refractive power between pre- and post-cycloplegia in hyperopic eyes were larger than those in myopic eyes (both *P* < 0.05). No significant difference was found in the change in posterior lens curvature and refractive power after cycloplegia in hyperopic and myopic eyes (*P* > 0.05).

**Conclusion:**

Anterior and posterior surfaces of the lens were flatter, and the refractive power was lower in the myopia group than in the hyperopia group. Myopic and hyperopic patients showed a tendency for lens flattening and refractive power decrease after cycloplegia. Hyperopic patients had more changes in anterior lens curvature and refractive power after cycloplegia.

## Highlights

- The mechanism of myopia is not yet clear.
- This study focuses on the changes of lens refractive power and curvature before and after cycloplegia in different people with myopia and hyperopia, to reveal the differences in lens physiological characteristics between them and provide a new direction for the exploration of the mechanism of myopia.

## Introduction

Myopia is a global public health concern. The World Health Organization predicts that 49.8% of the population will suffer from myopia by 2050 (1–3). Compared with the rapid growth trend of myopia, the mechanism of myopia is still not fully understood, which poses a great challenge to its prevention and control. During the development of human vision, the cornea and lens are constantly changing. When the cornea, lens, and axial development maintain a dynamic balance, the human eye can maintain a state of emmetropia (4–6). Once a part of this balance develops abnormally and its integrity is destroyed, it may manifest as myopia and other ametropia. Therefore, it is of great significance to observe the difference in eyeball structure under various refractive states to understand the mechanism of myopia.

There are several theories about the occurrence and development of myopia; of these, the theory of periretinal hyperopia defocus is widely accepted. Animal experiments have shown that myopic defocus may induce hyperopia, while hyperopia defocus may induce myopia; the latter may have a stronger inducing effect on myopia (7–9). In the process of myopia, the anterior chamber will deepen, and the lens will become thinner. When the curvature radius of the lens increases and its refractive power decreases, the object falls behind the retina through the refractive stroma and forms a hyperopia defocus, which may lead to the occurrence of myopia (10–20).

At present, two methods for measuring lens refractive power and deformation are widely accepted and are as follows: (1) measurement of the structural parameters such as ACD and AL of the eyeball and (2) measurement of the curvature radius of the anterior and posterior surfaces of the cornea and lens. The refractive power of the lens is calculated from the measurement results in both methods (21–24).

Few instruments have been used to directly measure the lens refractive power. In this study, all parameters were measured using an IOLMaster 700. The parameters of the cornea and lens were directly evaluated and indirectly calculated using MATLAB software using the fitting and formula methods, respectively; these were employed to evaluate changes in the ocular biological parameters between hyperopia, myopia, and pre- and post-cycloplegia with tropicamide.

The aim of this study was to explore the possible pathogenesis and biological basis of myopia by comparing the changes in ocular structure under different refractive states as well as to explore the effect of cycloplegia on the anterior segment and lens.

## Materials and methods

### Subjects

This study was conducted between August and October 2018 at the Ophthalmology Department of the Eye and ENT Hospital, Shanghai, China. The study protocol followed the principles of the Declaration of Helsinki and was approved by the ethics committee of the Eye and ENT Hospital of Fudan University. All participants provided written informed consent after the purpose of the study was explained to them in detail.

All subjects had the following qualities: (1) age between 6 and 12 years; (2) refractive spherical equivalent (RSE) from −6.00 diopter (D) to +6.00 D, with astigmatism not more than 1.5 D after cycloplegia; (3) best corrected visual acuity not < 0.5 in LogMar, with good fixation to the target; and (4) clear cornea and crystalline lens without visible opacity under slit-lamp examination. The exclusion criteria were as follows: (1) history of serious dry eye, corneal opacity, congenital cataract, glaucoma, uveitis, strabismus, and nystagmus; (2) history of using 0.01% atropine eye drops or orthokeratology for controlling myopia progression; and (3) history of any eye trauma and surgery. All subjects were classified into two groups according to the RSE after cycloplegia: (1) myopic group with RSE from −6.00 D to −0.50 D and (2) hyperopic group with RSE from +0.5 D to +6.00 D.

Potential candidates received objective refractions thrice. The average values were subsequently obtained. IOL Master 700 measurements were then performed. Subject attributes were as follows: (1) the candidate was seated; (2) the chin was placed on the chinrest; (3) the forehead rested, and (4) the eyes were looking forward. Before the measurement, the subject was instructed to blink thrice and then to look straight ahead. All measurements were conducted in the automatic mode with an active enhanced scan display. A valid measurement was completed when all parameters displayed a “green check” status on the display interface. After IOLMaster 700 examination, the subjects received tropicamide 1% for cycloplegia every 5 min, three times overall. Thirty minutes after the last administration, objective and subjective refractions as well as IOLMaster 700 measurements were performed. Only one eye from each subject was included in this study.

The IOLMaster 700 is a non-contact device that uses swept-source OCT technology at a 1,055-μm wavelength. B-scans were generated to determine the eye biometry. All measurements were the average values of the three scans for each of the six meridians. The axial length (AL), anterior chamber depth (ACD), lens thickness (LT), and central corneal thickness (CCT) measurements were based on the swept-source technology. Corneal curvature measurements were based on reflected light spots on the anterior corneal surface. Pupil diameter (PD) and white-to-white (WTW) measurements were based on scleral and iris images.

The calculation of lens curvature and lens power were as follows:

We set the corneal refractive index as *n*_0_ = 1.3375, the aqueous refractive index as *n*_1_ = 1.3333, the lens refractive index as *n*_2_ = 1.4160, the vitreous refractive index as *n*_3_ = 1.3333, the corneal power as Km, and the lens thickness as LT. The specific values of anterior chamber depth, pupil diameter, and central lens thickness were obtained using IOLMaster 700. The original image of the 6-mm area of the lens layer on the optic axis was obtained; the original image was reconstructed to obtain a scanning tomogram of the optic axis area. The anterior and posterior surfaces of the cornea, anterior and posterior surfaces of the lens, and boundary of the pupil were marked using semi-automatic software. The corresponding interface was fitted and described according to the tomogram; the fitting curve of the anterior and posterior surface of the lens was obtained; and the anterior (AL Curv) and posterior lens surface curvature radius values (PL Curv) were obtained using the MATLAB image processing software. This program utilized an algorithm as follows:

The anterior corneal curvature = (*n*_0_ – 1)/Km

AL Curv = the anterior corneal curvature × lens anterior surface curvature pixel/the anterior corneal curvature pixel

PL Curv = the anterior corneal curvature × lens posterior surface curvature pixel/the anterior corneal curvature pixel

The refractive power of the front surface of the lens:

AL Power = (*n*_2_ – *n*_1_)/AL Curv.

The refractive power of the posterior surface of the lens:

PL Power = (*n*_3_ – *n*_2_)/PL Curv.

The total refractive power of the lens:

TL Power = AL Power + PL Power – LT × AL Power × PL Power/*n*_2_.

### Statistical analysis

All statistical analyses were performed using MedCalc Statistical Software version 11.0 (MedCalc Software Inc., Mariakerke, Belgium). Statistical significance was set at *P* < 0.05. The distribution of all datasets was analyzed for normality using Kolmogorov–Smirnov tests. A paired *t*-test was applied to compare the ocular lens parameters obtained pre- and post-cycloplegia in the two groups. An independent sample *t*-test was used to compare ocular lens parameters between hyperopic and myopic eyes.

## Results

One hundred eyes of 100 participants (including 50 boys) were included in this study. In the myopic group, 50 subjects (including 25 boys and 25 right eyes) were enrolled with a mean age of 9.20 ± 1.69 years old. On the other hand, the hyperopic group included 50 subjects (including 25 boys and 25 right eyes) with a mean age of 8.20 ± 1.67 years old. Table 1 shows the spherical equivalent of objective and subjective refractions in the hyperopic and myopic groups pre- and post-cycloplegia.

**Table 1:** Spherical equivalent of objective and subjective refractions in hyperopic and myopic eyes.

**Refraction**	**Mean ±SD**	**Min**	**Max**
**Hyperopic eyes (*****n*** **=** **50)**
SEQ1	2.438 ± 1.594	−0.25	6.250
SEQ2	3.430 ± 1.693	0.375	6.250
SEQ3	3.465 ± 1.622	0.500	6.000
**Myopic eyes (*****n*** **=** **50)**
SEQ1	−3.135 ± 1.491	−6.125	−0.750
SEQ2	−2.837 ± 1.526	−7.125	−0.750
SEQ3	−2.696 ± 1.425	−6.000	−0.500

SEQ1, spherical equivalent of objective refraction before cycloplegic; SEQ2, spherical equivalent of objective refraction after cycloplegic; SEQ3, spherical equivalent of subjective refraction after cycloplegic; SD, standard deviation.

Table 2 shows the changes of ocular lens parameters in hyperopic eyes between pre- and post-cycloplegia obtained using the IOLMaster 700. The AL and PL Curv_H_ post-cycloplegia were 1.490 ± 1.583 mm and 0.260 ± 0.385 mm larger than pre-cycloplegia, respectively (both *P* < 0.001). The AL, PL, and TL Power_H_ were 1.010 ± 1.150 D, 0.664 ± 0.974 D, and 1.617 ± 1.648 D lower than pre-cycloplegia, respectively (all *P* < 0.001).

**Table 2:** Changes of ocular lens parameters between pre- and post-cycloplegia obtained by IOLMaster 700 in hyperopic eyes (*n* = 50).

	**Mean ±SD**	**t-values**	***P*-values**
ΔAL Curv_H_ (mm)	1.490 ± 1.583	6.655	< 0.001
ΔPL Curv_H_ (mm)	0.260 ± 0.385	4.761	< 0.001
ΔAL Power_H_ (D)	−1.010 ± 1.150	−6.212	< 0.001
ΔPL Power_H_ (D)	−0.664 ± 0.974	−4.825	< 0.001
ΔTL Power_H_ (D)	−1.617 ± 1.648	−6.938	< 0.001

Table 3 shows the changes of ocular lens parameters between pre- and post-cycloplegia in myopic eyes. The AL and PL Curv_M_ after cycloplegia were 0.766 ± 1.127 and 0.180 ± 0.421 mm larger than those before cycloplegia, respectively (both *P* < 0.001). The AL, PL, and TL Power_M_ after cycloplegia were 0.366 ± 0.501 D (*P* < 0.001), 0.383 ± 0.893 D (*P* = 0.004), and 0.728 ± 1.113 D (*P* < 0.001) lower than the values before cycloplegia, respectively.

**Table 3:** Comparison of ocular lens parameters between pre- and post-cycloplegia obtained by IOLMaster 700 in myopic eyes (*n* = 50).

	**Mean ±SD**	**t-values**	***P*-values**
ΔAL Curv_M_ (mm)	0.766 ± 1.127	4.832	< 0.001
ΔPL Curv_M_ (mm)	0.180 ± 0.421	3.019	0.004
ΔAL Power_M_ (D)	−0.366 ± 0.501	−5.105	< 0.001
ΔPL Power_M_ (D)	−0.383 ± 0.898	−3.011	0.004
ΔTL Power_M_ (D)	−0.728 ± 1.113	−4.637	< 0.001

Table 4 shows the ocular lens parameters between hyperopic and myopic eyes obtained using the IOLMaster 700. The AL and PL Curv in myopic eyes without cycloplegia were 1.468 ± 0.352 mm and 0.391 ± 0.124 mm larger, respectively, than those in hyperopic eyes without cycloplegia (both *P* < 0.001). The AL, PL, and TL powers in myopic eyes without cycloplegia were 1.113 ± 0.222, 0.969 ± 0.228, and 1.914 ± 0.379 D lower than those in hyperopic eyes without cycloplegia, respectively (all *P* < 0.001). The AL and PL Curv in myopic eyes after cycloplegia were 0.788 ± 0.236 mm and 0.301 ± 0.181 mm larger, respectively, than those in hyperopic eyes after cycloplegia (both *P* < 0.001). The AL, PL, and TL powers in myopic eyes after cycloplegia were 0.349 ± 0.144 D (*P* = 0.018), 0.629 ± 0.154 D (*P* < 0.001), and 0.998 ± 0.282 D (*P* < 0.001) lower than those in hyperopic eyes after cycloplegia, respectively.

**Table 4:** Ocular lens parameters of obtained by IOLMaster 700 between hyperopic and myopic eyes.

**Parameters**	**Mean ±SE**	***t*-values**	***P*-values**
**Pre-cycloplegic**
AL Curv_pre − M−H_ (mm)	1.468 ± 0.352	4.276	< 0.001
PL Curv_pre − M−H_ (mm)	0.391 ± 0.124	4.012	< 0.001
AL Power_pre − M−H_ (D)	−1.113 ± 0.222	−4.258	< 0.001
PL Power_pre − M−H_ (D)	−0.969 ± 0.228	−4.012	< 0.001
TL Power_pre − M−H_ (D)	−1.914 ± 0.379	−4.978	< 0.001
**Post-cycloplegic**
AL Curv_post − M−H_ (mm)	0.788 ± 0.236	2.862	< 0.001
PL Curv_post − M−H_ (mm)	0.301 ± 0.181	2.989	< 0.001
AL Power_post − M−H_ (D)	−0.349 ± 0.144	−2.421	0.018
PL Power_post − M−H_ (D)	−0.629 ± 0.154	−3.238	< 0.001
TL Power_post − M−H_ (D)	−0.998 ± 0.282	−3.573	< 0.001

**Figure 1:**
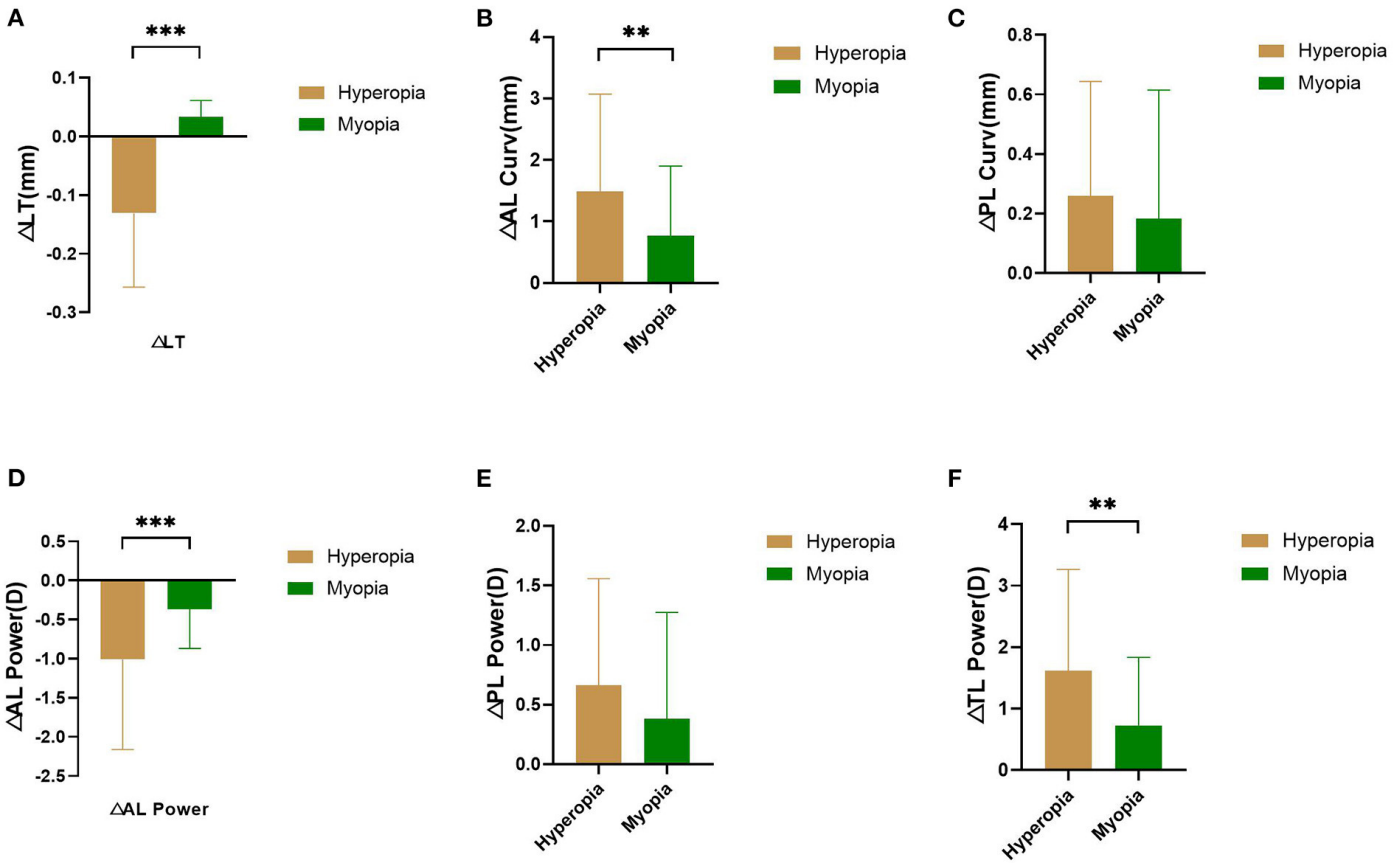
Comparison of changes of LT **(A)**, AL Curv **(B)**, PL Curv **(C)**, AL Power **(D)**, PL Power **(E)**, and TL Power **(F)** between pre- and post- cycloplegia in hyperopic and myopic eyes. **P < 0.01, ***P < 0.001.

Figure 1 shows the comparison of changes in ocular lens parameters between pre- and post-cycloplegia obtained by IOLMaster 700 in hyperopic and myopic eyes obtained using the IOLMaster 700. ΔLT were 0.131 ± 0.126 and −0.033 ± 0.028 in hyperopic and myopic eyes, respectively. The difference between the two was statistically significant (*P* < 0.001). ΔAL Curv were −1.490 ± 1.582 and −0.766 ± 1.127 in hyperopic and myopic eyes, respectively. The difference between the two was statistically significant (*P* = 0.010). ΔPL Curv were −0.259 ± 0.385 and −0.180 ± 0.421 in hyperopic and myopic eyes, respectively. There was no statistically significant difference between the two (*P* = 0.358). ΔAL Power were 1.010 ± 1.150 and 0.366 ± 0.501 in hyperopic and myopic eyes, respectively. The difference between the two was statistically significant (*P* < 0.001). ΔPL Power were 0.664 ± 0.893 and 0.383 ± 0.898 in hyperopic and myopic eyes, respectively. There was no statistically significant difference between the two (*P* = 0.135). ΔTL power was 1.617 ± 1.648 and 0.728 ± 1.113 in the hyperopic and myopic eyes, respectively. The difference between the two was statistically significant (*P* = 0.002).

## Discussion

Several theories have attempted to explain the causes of myopia, including the choroidal ischemia, hyperopia defocus, accommodation, genetic, and form deprivation theories; all of which can be explained by different layers, such as blood supply, retinal imaging, accommodation, and vision development (25–29). As a global concern, the occurrence of myopia may be related to all of the above factors. This study focused on the comparison of lens refractive power in children with different refractive states, and further explore the changes of lens morphology and optical properties after cycloplegia with tropicamide, to propose new directions and ideas for mechanism of myopia

In the development of emmetropia, it has been proven that there are a series of structural eye changes, such as axial growth as well as lens thinning, surface flattening, and refractive power weakening (10, 12, 16, 20, 26). In this study, the AL, PL, and TL refractive powers of children with myopia under natural pupils were significantly lower than those with hyperopia. Correspondingly, the AL and PL curvature radius of children with myopia under natural pupils were significantly higher than those with hyperopia. Li et al. (16) showed that the curvature radius of the anterior and posterior surfaces of the lens in myopic patients was larger than that in emmetropic eyes, i.e., the lens was flatter. Furthermore, He et al. (10) showed that the lens in myopic patients was thinner, i.e., the lens refractive power was weaker. These results are in agreement with our results. Given the condition that the lens becomes thinner, its anterior and posterior surfaces become flat, and its refractive power decreases during the development of vision, object images will fall behind the retina, i.e., hyperopia defocus, thereby inducing axial growth and leading to myopia (17, 18). This study confirmed that the anterior and posterior surfaces of the lens in myopic children are flatter than those in hyperopia; the refractive power of the lens is smaller than that of hyperopia. The difference between myopia and hyperopia in the above aspects may indicate the importance of ocular structure changes in the process of myopia.

In terms of the choice of cycloplegic drugs, the patients included in this study are all patients who are regularly followed up in our hospital. Although previous studies have shown that tropicamide may be a relatively weak cycloplegic drug (30), considering that the patients included in this study used tropicamide for cycloplegia in the past, this study finally chose tropicamide for cycloplegia. In our study, the curvature radius of the anterior and posterior surfaces of the lens increased; its refractive power decreased after cycloplegia with tropicamide. Studies have shown that cycloplegia has a great influence on the measurement of lens refractive power (31, 32). These changes are related to ligament tension and stretching of the lens, which is consistent with Helmholz's classical eye regulation theory. This study further verified the above conclusions, and more accurately measured the changes in the refractive power and curvature radius of the anterior and posterior surfaces of the lens, which further described the changes in morphology and function of the lens under cycloplegia. The results demonstrated that the changes in LT, AL curvature radius, AL refractive power, and TL refractive power in children with hyperopia between pre- and post-cycloplegia were greater than those in children with myopia, whereas there was no significant difference in the changes in PL curvature radius and PL refractive power between pre- and post-cycloplegia. This finding may explain why the hyperopic group had more and stronger accommodation than the myopic group. It is worth noting that the effect of cycloplegia on the posterior surface of the lens seems to be not as significant as that on the anterior surface. The PL refractive power in the myopic group was significantly lower than that in the hyperopic group, and the PL curvature radius was significantly greater than that in the hyperopic group, no matter before or after cycloplegia. However, after cycloplegia, there was no significant difference in the changes of PL refractive power (*P* = 0.135) and PL curvature radius (*P* = 0.358) between the two groups. This may suggest that in the process of myopia, the main manifestation of the lens is the decrease of the “elasticity” of the anterior surface, although the morphology of the entire lens has changed.

In this study, the IOLMaster 700 was used to measure the different refractive states of children aged 6–12. It was also used to analyze the differences and to calculate the curvature and refractive power of the anterior and posterior surfaces of the lens, which is helpful in understanding the mechanism of myopia. At the same time, by analyzing the changes in lens parameters after cycloplegia, this study may be helpful in exploring the differences between lenses in varying refractive states. It may also aid in understanding the process of cycloplegia and accommodation.

## Limitation

This was a cross-sectional study; we cannot understand the specific changes in the lens morphology and refractive power during the development of myopia. In addition, this study did not use cyclopentolate for cycloplegia, which may have an impact on the examination after cycloplegia. Therefore, further cohort studies are warranted.

## Conclusion

The anterior and posterior surfaces as well as the refractive power of the lens in the myopia group were flatter and lower as compared with the hyperopia group, respectively. Both myopic and hyperopic patients showed a tendency for lens flattening and refractive power weakening after cycloplegia, which is consistent with existing theories. In addition, hyperopic patients had more changes in the above indicators before and after cycloplegia, which may explain the stronger accommodative power of hyperopic patients. In the process of myopia, the change of the anterior surface of the lens may be more important than the posterior surface, which is also the direction of follow-up research.

## Data availability statement

The raw data supporting the conclusions of this article will be made available by the authors, without undue reservation.

## Ethics statement

The studies involving human participants were reviewed and approved by the Ethics Committee of the Eye and ENT Hospital of Fudan University. Written informed consent to participate in this study was provided by the participants' legal guardian/next of kin.

## Author contributions

JS is responsible for writing the article. JS and YH are responsible for patient follow-up and data entry. JS, YH, and YW complete the calculation of crystal diopter. JH and XZ provide opinions and suggestions in the research and analysis. YH and XQ are the corresponding authors of this paper and lead the design of this study. All authors contributed to the article and approved the submitted version.
